# Mechanical and Thermal Properties of Geopolymer Foams (GFs) Doped with By-Products of the Secondary Aluminum Industry

**DOI:** 10.3390/polym14040703

**Published:** 2022-02-11

**Authors:** Roberto Ercoli, Dorota Laskowska, Van Vu Nguyen, Van Su Le, Petr Louda, Piotr Łoś, Justyna Ciemnicka, Karol Prałat, Alberto Renzulli, Eleonora Paris, Matteo Basilici, Cezary Rapiejko, Katarzyna Ewa Buczkowska

**Affiliations:** 1 Department of Pure and Applied Sciences, University of Urbino Carlo Bo, Via Ca’ Le Suore 2/4, 61029 Urbino, Italy; alberto.renzulli@uniurb.it; 2 Department of Mechanical Engineering, Koszalin University of Technology, Śniadeckich 2, 75-453 Koszalin, Poland; dorota.laskowskapl@gmail.com; 3 Department of Material Science, Faculty of Mechanical Engineering, Technical University of Liberec, Studentska 2, 461 17 Liberec, Czech Republic; nguyen.van.vu@tul.cz (V.V.N.); su.le.van@tul.cz (V.S.L.); petr.louda@tul.cz (P.L.); piotr.los@tul.cz (P.Ł.); katarzyna.ewa.buczkowska@tul.cz (K.E.B.); 4 Faculty of Civil Engineering, Mechanics and Petrochemistry, Institute of Civil Engineering, Warsaw University of Technology, I. Łukasiewicza 17, 09-400 Płock, Poland; justyna.ciemnicka@pw.edu.pl (J.C.); karol.pralat@pw.edu.pl (K.P.); 5 Geology Division, School of Science and Technology, University of Camerino, Via Gentile III da Varano, 62032 Camerino, Italy; eleonora.paris@unicam.it (E.P.); matteo.basilici@unicam.it (M.B.); 6 Department of Materials Technology and Production Systems, Faculty of Mechanical Engineering, Lodz University of Technology, Stefanowskiego 1/15, 90-001 Lodz, Poland; cezary.rapiejko@p.lodz.pl

**Keywords:** geopolymer foam, hydrogen, secondary aluminum, by-products recycling

## Abstract

The article deals with the investigation of geopolymer foams (GFs) synthesized using by-products coming from the (i) screening-, (iv) pyrolysis-, (iii) dust abatement- and (iv) fusion-processes of the secondary aluminum industry. Based on principles of the circular economy to produce new technological materials, the experimental study involves industrial by-products management through the recovery, chemical neutralization, and incorporation of these relatively hazardous waste into the GFs. The geopolymeric matrix, consisting of metakaolin (MK) and silica sand (SA) with a 1:1 wt.% ratio, and chopped carbon fibers (CFs, 1 wt.% MK), was doped with the addition of different aluminum-rich industrial by-products with a percentage from 1 to 10 wt.% MK. The gas (mainly hydrogen) produced during the chemical neutralization of the by-products represents the foaming agents trapped in the geopolymeric structure. Several experimental tests were carried out to characterize the mechanical (flexural, compressive, and Charpy impact strengths) and thermal properties (thermal conductivity, and diffusivity, and specific heat) of the GFs. Results identify GFs with good mechanical and thermal insulation properties, encouraging future researchers to find the best combination (for types and proportions) of the different by-products of the secondary aluminum industry to produce lightweight geopolymer foams. The reuse of these industrial by-products, which according to European Regulations cannot be disposed of in the landfill, also brings together environmental sustainability and safe management of hazardous material in workplaces addressed to the development of new materials.

## 1. Introduction

Geopolymers are engaging materials due to their favorable properties such as high mechanical strengths, low thermal conductivity, high thermal stability, a good fire and acid resistance [1,2,3,4,5,6,7,8,9,10,11]. In addition, low-density geopolymers have several advantages and are materials with applications in many fields. In particular, geopolymer foams (GFs) can be used as building materials, thermal insulators, adsorbents, catalysts, and fillers [12,13,14]. They are specially designed for insulation and fire resistance due to their low thermal conductivity [15,16,17]. Moreover, they also exhibit several advantages from an economic and ecological point of view compared to the Portland cement [18].

The present study concerns the sustainable recovery and reuse of by-products from the secondary aluminum industry as foaming agents trapped in the geopolymeric structure. This process is achievable by the chemical neutralization (oxidation) of the metallic aluminum-rich materials during the geopolymerization. The interaction between the industrial by-products and the binder of the geopolymer (metakaolin and alkaline activator) will produce gas (mostly hydrogen), forming bubbles responsible for the low density of the GFs. Regarding the waste management of secondary aluminum production, it is well known that industries worldwide produce large amounts of by-products since the recycling process requires pre-treatments to obtain suitable scrap for melting, refining, and casting. The primary by-products from the secondary aluminum industry come from mixing, comminution, screening, pyrolyzing, aluminum melting, fumes abatement collected by the decorator, and centrifugal dust collector.

The European regulations classify the aluminum-rich waste materials as special hazardous wastes, which can develop flammable gases and form explosive mixtures with air (HP4-HP14 hazard class and 100323* EWC). Reasonably, these industrial by-products derived from the secondary aluminum industry exhibit a non-compliant eluate to facilities non-hazardous waste landfill, under the criteria established for their admissibility. These by-products are hazardous because of their high amounts of metals (primary aluminum) that drive reactions associated with a potential source of hydrogen release [19].

The reuse of industrial by-products derived from the recycling processes of secondary aluminum industries represents an essential response to the need to create sustainable economic growth, grounded on decreasing natural resources and minimizing waste output. Nowadays, the only three digestion solutions of the non-reusable materials such as the aluminum scraps are (i) aerobic/anaerobic bio-oxidation cold systems, (ii) gasification, pyrolysis, or hot incineration systems, and (iii) the disposal of in landfills, that designates the main resolution [20,21,22]. To avoid these methods, we discuss virtuous alternatives of waste reuse, such as geopolymer foams having good thermal and sound insulation properties, reducing heat loss, and enhancing soundproofing in buildings [23]. Moreover, one of the most promising applications of geopolymers is their use as waste encapsulating matrices. These binders can activate several chemical and physical immobilization mechanisms for a wide variety of inorganic waste materials. Several studies have investigated the immobilization of cations, mainly heavy metals or even radioactive wastes, and specifically dust from filters of the secondary aluminum industry as raw materials to produce geopolymer foams [24,25,26,27,28,29,30,31,32].

**Figure 1:**
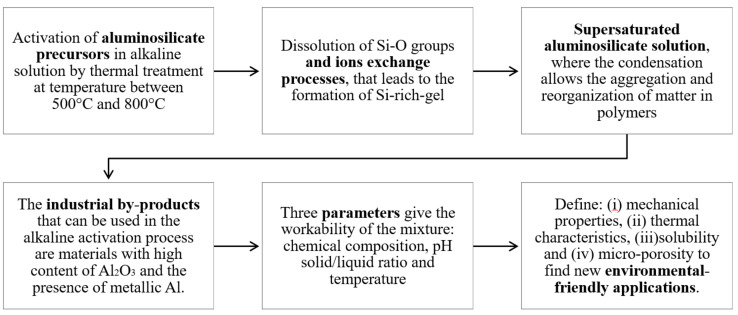
Scheme of the geopolymerization process by using the industrial by-products of the secondary aluminum industry as foaming agents.

The process of converting waste materials and structurally reorganizing aluminosilicate precursors and alkaline activators into geopolymers is called geopolymerization [33,34]. The aluminosilicates disaggregation occurs under the same experimental conditions of chemical neutralization, forming a supersaturated aluminosilicate solution and geopolymers condensation. It strongly depends on the chemical composition, solid/liquid ratio, pH, and thermodynamics [35]. During this process (Figure 1), a silica gel forms rearrange itself, creating a three-dimensional structure [36,37,38]: aluminum and silicon ions are tetrahedrally coordinated mine while alkali balances the electrical charge associated with the ion exchanges.

## 2. Materials and Methods

### 2.1. Starting Materials

The inorganic two-component aluminosilicate binder (commercial name: Bausik LK), (České lupkové závody, a.s., Nové Strašecí, Czech Republic) [39] is a two-component aluminosilicate binder based on metakaolin (hereafter MK, part A), (commercial name: Mephisto L05), (grain size D_50_ = 3 µm, D_90_ = 10 µm) activated by an aqueous alkaline activator (part B). The mixing ratio of these two components was taken out according to the manufacturer requirements. In preparing the binder mixture based on the inorganic polymer, five parts by weight of part A and four parts of B (activator) are usually used. The silica sand (hereafter SA, ST 01/06), (Sklopísek Střeleč, a.s., Újezd pod Troskami, Czech Republic), (D_50_ = 0.44 mm, D_90_ = 0.63) [40] was used as aggregate. Chopped carbon fibers with an elastic module up to 230 GPa and tensile strength of 3500 MPa [41,42,43,44,45] were used as reinforcing materials. Table 1 shows the chemical composition of the raw materials used in this experiment to produce the geopolymer-based matrix.

Various aluminum-rich by-products (Table 2) were used as additives to foam the geopolymers. The studies of the starting materials were conducted with specific analytical techniques to determine the chemical content subsequently indicated and for the planning of laboratory experiments. The chemical analyses of the by-products of the secondary aluminum industry were performed by ICP-MS with near-total multi-acids (hydrofluoric, nitric, and perchloric acids) digestion at Actlabs (Ancaster, ON, Canada). After the digestion and dehydration, only specific species of the sample were brought into solution using aqua regia and analyzed with ten duplicates and eight reference materials through Perkin Elmer Sciex ELAN ICP-MS.

**Figure 2:**
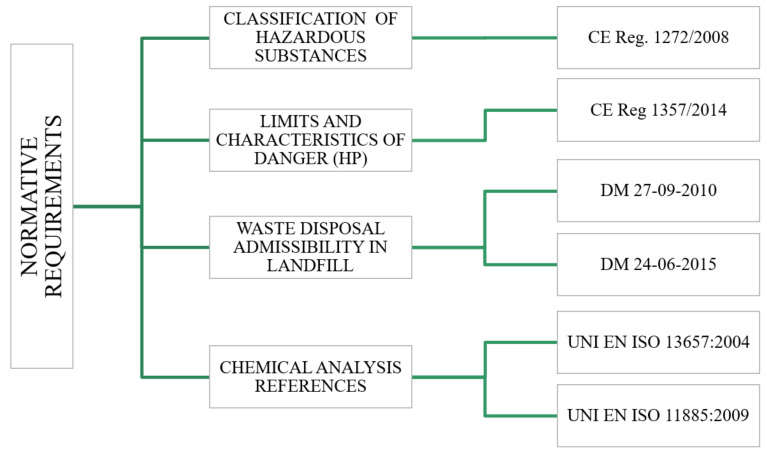
Normative requirements for the classifications of hazardous substances, limits, and characteristics of danger (HP), waste disposal admissibility in landfills, and chemical analysis references.

The data processing enabled a quantitative assessment of the dangerous compounds in the aluminum processing slags, which are critical when reused [46,47]. The samples were classified under the normative requirements (Figure 2) of the Decree of environmental assessments and authorizations n.31/VAA (30 April 2015) [48], which were used by the European industries to issue the integrated environmental authorization (AIA) (EU directive 2010/75 and Legislative Decree 152/2006) [49,50], on the environmental safety and pollution control. The normative requirements provide the classification of hazardous substances on the CE Reg. 1272/2008 [51] and limits and characteristics of danger (HP) on the CE Reg. 1375/2014 [52].

**Figure 3:**
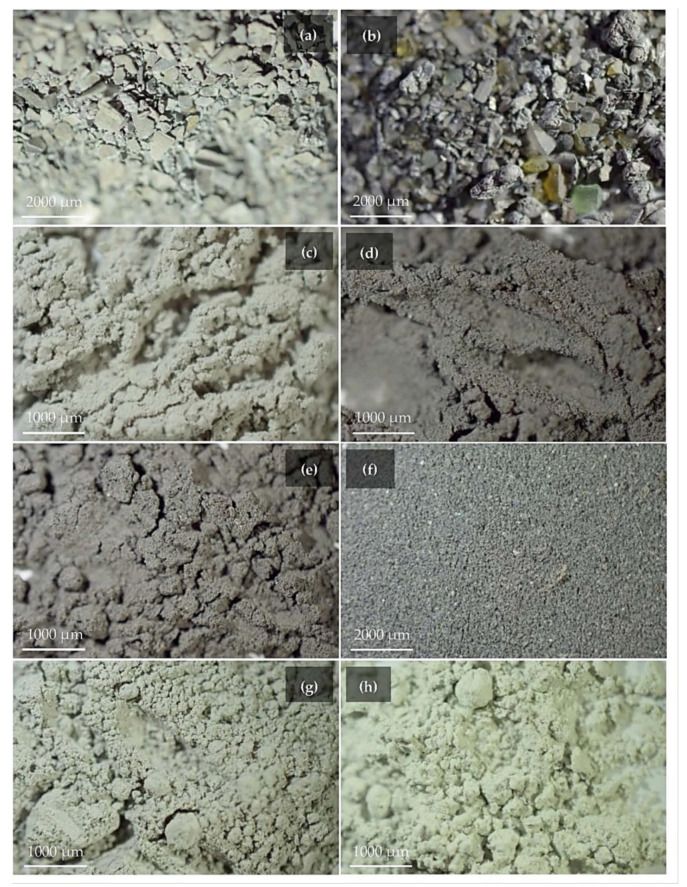
Photos of by-products of the secondary aluminum industry: V.FG (**a**) and V.UBC (**b**): screening; D.FG (**c**) and D.UBC (**d**): pyrolysis; C.FG (**e**) and C.UBC (**f**): abatement dust; FF.FG (**g**) and FF.UBC (**h**): fusion slags.

A macroscopic overview of the aluminum-rich by-products is given in Figure 3. The materials used as fillers into the geopolymers derive from the main processes of the secondary aluminum industry: (i) screening process, (ii) pyrolysis process, (iii) fusion process. FG and UBC acronyms are from coarse-grained domestic appliance scrapes and urban beverage cans, the primary materials used for recycling.

**Figure 4:**
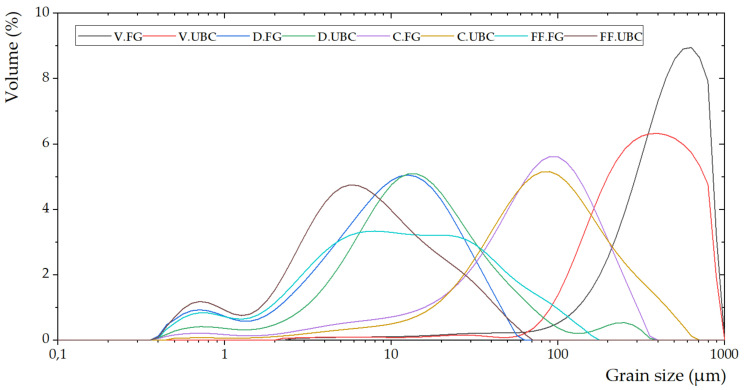
Grain size modal curve of the aluminum-rich by-products.

The powder X-ray analyses of the aluminum-rich by-products were determined with a Bruker D8 Advance diffractometer at CRI.ST (Centro di Servizi di CRIstallografia STrutturale, Florence, Italy), and a Philips X’Change PW1830 powder diffractometer at University of Urbino (Urbino, Italy). The grain size analyses (Figure 4) were performed through a Laser beam particle analysis (Hydro 2000MU analyzer, University of Milano-Bicocca, Milan, Italy).

V.FG (2.52–893.37 µm) and V.UBC (2.00–893.37 µm) (Figure 3a,b) represent by-products from the screening process of the secondary aluminum industry. The mineralogical phases are metallic aluminum and rutile in V.FG, whereas metallic aluminum, quartz, periclase, and carlinite are in V.UBC. The aluminum content is 125,468 ppm and 180,638 ppm, respectively. 

D.FG (0.40–56.37 µm) and D.UBC (0.40–355.66 µm) (Figure 3c,d) are produced during the pyrolysis process. Their aluminum contents are 32,204 ppm and 40,198 ppm. Aluminum, portlandite, rutile, and CaClOH are the main mineralogical phases detected within the two materials. 

C.FG (0.40–355.66 µm) and C.UBC (0.45–632.46 µm) (Figure 3e,f), (dust materials caught from the cyclones) present aluminum contents of 73,296 ppm and 62,333 ppm. The mineralogical phases are aluminum calcite, rutile, graphite, ankerite in C.FG, and zinc in C.UBC. 

The industrial by-products from the secondary aluminum fusion process, FF.FG (0.40–158.87 µm) and FF.UBC (0.40–63.25 µm) (Figure 3g,h), have an aluminum content of 14,549 ppm and 6636 ppm. The mineralogical pattern is metallic aluminum, halite, sylvite, and portlandite.

### 2.2. Experimental Procedure for the Geopolymer Synthesis

Several geopolymers were synthesized to investigate the influence of the aluminum-rich by-products on several physical properties: flexural strength, compressive strength, Charpy impact strength, thermal conductivity, specific heat, and thermal diffusivity. 

For this purpose, metakaolin (MK), (Al_2_O_3_ 40.1 wt.%; SiO_2_: 54.1 wt.%) have been used during the alkaline activation process as precursor materials, using a potassium hydroxide aqueous solution (A) (pH 11) [53,54]. In addition, chopped carbon fibers, which show evidence to increase the mechanical properties of the materials [55], are employed in the REF-2 geopolymer and in the geopolymer foams where aluminum waste materials represent additives for foaming.

The previously described aluminum-rich by-products would play the role of foaming agent, generating H_2_-enriched gas pockets inside the geopolymer structure and making the material more porous and therefore lighter. The foaming process regards the aluminum and alkaline aqueous solution interaction, where the potassium hydroxide reacts, forming tetra hydroxy aluminate (III) and hydrogen gas, and aluminum undergoes oxidation. The primary reaction involved is described by the Reaction (1):

(1) $$2\mathrm{Al}+2\mathrm{KOH}+6H_{2}O\to 2\mathrm{KAl}{\left(\mathrm{OH}\right)}_{4}+3H_{2}\;$$

**Figure 5:**
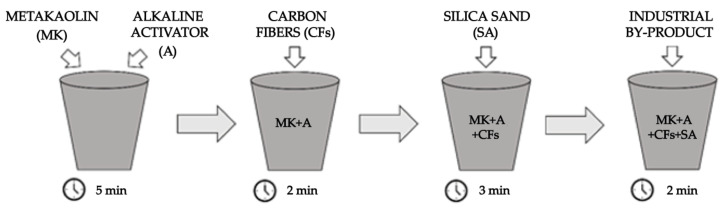
The preparation process of the geopolymer foams.

The experimental procedure reported in Figure 5 shows how the raw materials were mixed to prepare all the references and geopolymer foams.

The metakaolin (MK) and alkaline activator (A) were mixed for about 5 min to obtain a homogenous mortar. Next, chopped carbon fibers (CFs) were added, mixing for 2 min. After that, silica sand (SA) was added and mixed for 3 min. Finally, each industrial by-products (marked as V.FG, V.UBC, D.FG, D.UBC, C.FG, C.UBC, FF.FG, or FF.UBC) were mixed for 2 min in order to prepare different GFs (Table 3).

After the mixing, the geopolymer mortar was decanted into molds with the dimension of 30 × 30 × 150 mm (for three-point bending test and compression test), 19 × 20 × 60 mm (for Charpy impact test), and 100 × 100 × 100 mm (for thermal analysis). These samples were covered using a polypropylene film and cured at room temperature for about 24 h. After that time, the samples were pulled out of the molds, wrapped again using a polypropylene film, and kept at room temperature for 28 days before being analyzed (standard EN 12390-3:2019) [56].

Two types of reference samples were used. The first, labeled as REF-1, was made by mixing metakaolin, alkaline activator, and silica sand, obtaining a composition of SiO_2_ 76.6 wt.%, Al_2_O_3_ 20.1 wt.%, Fe_2_O_3_ 0.55 wt.%, K_2_O 0.40 wt.%, TiO_2_ 0.9 wt.%, CaO 0.07 wt.% and MgO 0.09 wt.%. The second reference sample, labeled as REF-2, was obtained by adding chopped carbon fibers. 

A name coding system was introduced to distinguish the geopolymers (Table 4). The first part indicates the type of the added industrial by-product (e.g., V.FG), the second its percentage (1, 2, 3, 5, and 10 wt.%) referred to the metakaolin (MK) (e.g., V.FG-1).

### 2.3. Methods for the Mechanical Tests

**Figure 6:**
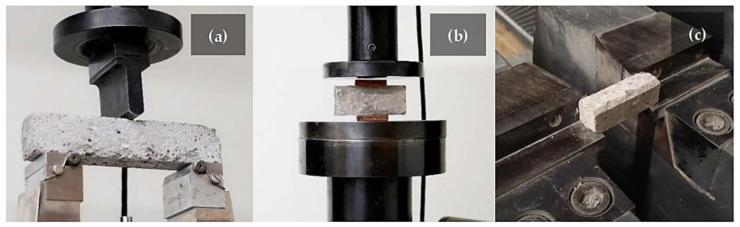
Laboratory techniques to carry out the three-point bending test (**a**), the compressive strength test (**b**), and the Charpy impact test (**c**).

The samples were cured for 28 days before being tested to characterize the mechanical properties of the GFs and the influence of the different by-products used as foaming agents. Figure 6 shows the three main laboratory instruments (at the Department of Material Science, University of Liberec, Liberec, Czech Republic) and techniques to carry out analyses for mechanical properties: (a) three-point bending test, (b) compressive strength test, (c) Charpy impact test.

The three-point bending tests were conducted using an INSTRON (Model 4202) Testing Machine (standard UNI EN 10002-1:2004) [57]. Tests were carried out on six 30 × 30 × 150 mm specimens (Figure 6a) at room temperature with a crosshead speed of 6.0 mm/min and a span length of 100 mm. The flexural strength ($\sigma_{f}$) was calculated by the Equation (2):

(2) $$\sigma_{f}=3L\;\frac{F_{\max}}{2{\mathrm{bh}}^{2}}\;\left(MPa\right)\;$$

where: $F_{\max}$—the maximum applied load indicated by the machine (N); L—the span length (mm); b—the width of the sample (mm); h—the depth of the sample (mm).

As for flexural strength determination, the compressive tests were performed employing the INSTRON (Model 4202) Testing Machine (standard EN 196-1:2016) [58]. The broken parts from the samples used in the bending test were used (Figure 6b). In this way, twelve samples with dimensions 30 × 30 × 30 mm were obtained for each composition. The tests were conducted at room temperature with a 6.0 mm/min crosshead speed. The compressive strength ($\sigma_{c}$) was obtained by the Equation (3):

(3) $$\sigma_{c}=\frac{F_{\max}}{A_{c}}\;\left(MPa\right)$$

where: $A_{c}$—the cross-sectional area of the sample (mm^2^).

The impact tests were carried out using a PIT-C Series Pendulum Impact Testing Machine (standard EN ISO 148-1:2010) [59] with a pendulum capacity of 150 J, energy losses compensation of 0.23 J, and estimated absorbed energy of 150 J. Six samples with the dimensions 19 × 20 × 60 (mm) were tested (Figure 6c). The tests were performed at room temperature. The impact strength ($\sigma_{i}$) was calculated by the Equation (4):

(4) $${\;\sigma }_{i}=\frac{E}{V}\;\left(MPa\right)$$

where: E—the absorbed energy indicated by the machine (J); V—the sample volume (mm^3^).

### 2.4. Methods for the Thermal Measurements

The thermal analyses were conducted at the Faculty of Civil Engineering, Mechanics and Petrochemistry, Warsaw University of Technology, Płock, Poland. After 28 days of curing, six measurements for each specimen were performed using the Isomet 2114 device (standard ASTM D5334-08) [60], a microprocessor-controlled commercial instrument with interchangeable probes.

A known heat source produced a wave propagating radially into the specimen. The dissipation of electrical energy generates the heat flow through the probes in direct contact with the material, and a serial port (RS-232C protocol) [61] records the signal. Semiconductor sensors at specific points on the materials sampled the temperature change in function of time: the temperature rises linearly with the logarithm of time [62,63,64,65].

According to the 2nd law of thermodynamics, the thermal conductivity $\left(\lambda \right)$ was determined by the Equation (5):

(5) $$\lambda =\frac{\mathrm{Qd}}{A\Delta T}\;\left(\frac{W}{mK}\right)\left\{\backslash \mathrm{displaystyle}\;\nabla T\right\}$$

where: Q—the amount of heat transferred, d—the distance between the two isotherms, A—the surface, and ΔT—the temperature gradient.

The specific heat capacity ($C_{p}$) is the heat needed to increase the temperature of 1 g of a substance by 1 °C and is given by:

(6) $$C_{p}=\frac{Q}{m\Delta T}\left(\frac{J}{KgK}\right)\left\{\backslash \mathrm{displaystyle}\;\nabla T\right\}$$

where: m—the mass.

The thermal diffusivity (α) quantifies the heat transfer rate of the material from the hot side to the cold side, and it was computed by the Equation (7):

(7) $$\alpha =\frac{\lambda }{{\rho C}_{p}}\left(\frac{mm^{2}}{sec}\right)\left\{\backslash \mathrm{displaystyle}\;\nabla T\right\}$$

where: ρ—the density of the geopolymer (obtained dividing the sample mass by volume—standard EN 1936:2006) [66].

## 3. Results and Discussion

### 3.1. Mechanical Properties

Mechanical properties are the most relevant parameters for evaluating geopolymer performances and understanding the applications [67,68]. The results of the three-point bending, compressive and Charpy impact strengths are shown in Table 4, where the reference samples are REF-1 and REF-2 (see Section 2.2).

We can observe a decrease in the bending and compressive strengths of REF-2 (compared to REF-1) where σ_f_ and σ_c_ are 6.25 ± 0.20 Mpa and 44.02 ± 2.08 Mpa, respectively. On the other hand, the Charpy impact strength value of REF-2 increases two times the REF-1 because of chopped carbon fibers, which, as mentioned, reinforce the geopolymer structure.

The reactivity of the industrial by-products used as fillers and foaming agents during the geopolimerization can be mainly attributed to the chemical composition (aluminum content), mineralogy, and grain size [69,70]. These features influence the physical and mechanical characteristics of the geopolymers thanks to the porosity formed during the aluminum oxidation [71,72,73,74,75]. 

It is highlighted that by adding the aluminum-rich by-products and increasing their percentage, the flexural and tensile strengths of the geopolymers decrease (Table 4) due to the gas bubbles formed in their structure during the consolidation process. On the other hand, most of the impact strengths data mainly increase.

**Figure 7:**
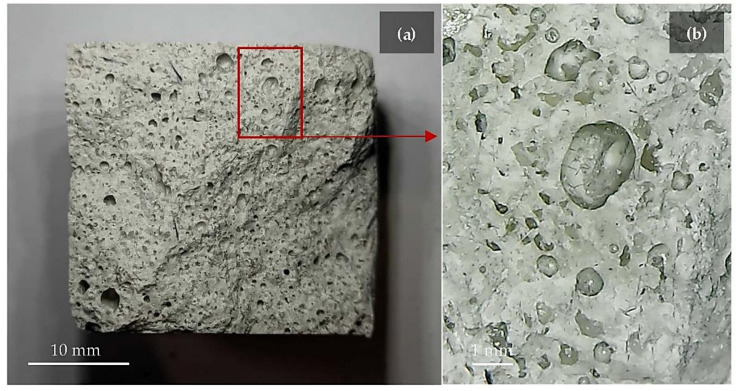
(**a**) FF.UBC-3 section (30 × 30 mm) and (**b**) magnified image of bubbles generated by the oxidation of the by-product.

Figure 7a,b illustrates the gas bubbles distribution of the geopolymer foam FF.UBC-3 that appear not homogeneous and characterized by different size holes. The areas of these bubbles were quantitatively estimated on the breaking section after the three-point bending tests by an open-source software analysis (ImageJ), applying a color threshold for the analysis. 13.2% of the total surface (900 mm^2^) consists of bubbles that, of course, define the overall geopolymer structure and shape the surface along which the break occurs.

#### 3.1.1. GFs with the Addition of the Aluminum-Rich By-Products of the Screening Processes

**Figure 8:**
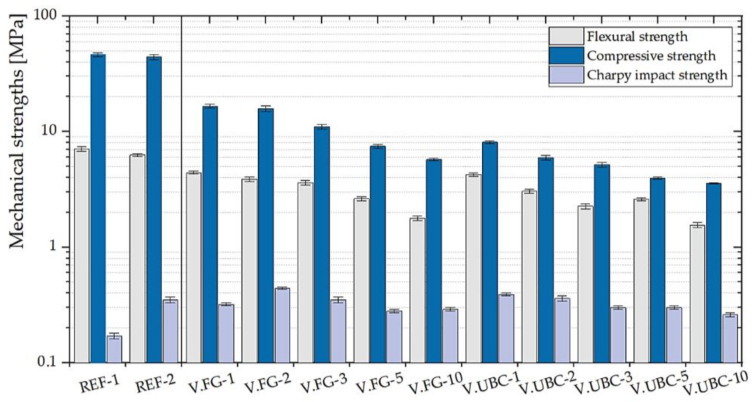
Mechanical properties of the geopolymer foams with the addition of various percentages of V.FG and V.UBC (1, 2, 3, 5, 10 wt.% of MK). Reference GPs (REF-1 and REF-2) are also shown.

The maximum detected values of the three-point bending and compressive strengths are identified in V.FG-1 (σ_f_ = 4.41 ± 0.11 MPa; σ_c_ = 16.56 ± 0.71 MPa) and V.UBC-1(σ_f_ = 4.25 ± 0.13 MPa; σ_c_ = 8.08 ± 0.2 MPa), following a decreasing trend by adding higher filler contents. The Charpy impact strength is improved than the reference geopolymers by adding 2 and 3 wt.% MK of V.FG (σ_i_ = 0.44 ± 0.01 MPa; 0.35 ± 0.02 MPa) and 1 and 2 wt.% MK of V.UBC (σ_i_ = 0.32 ± 0.01 MPa; 0.4 ± 0.01 MPa) (Figure 8).

#### 3.1.2. GFs with the Addition of the Aluminum-Rich By-Products of the Pyrolysis Processes

**Figure 9:**
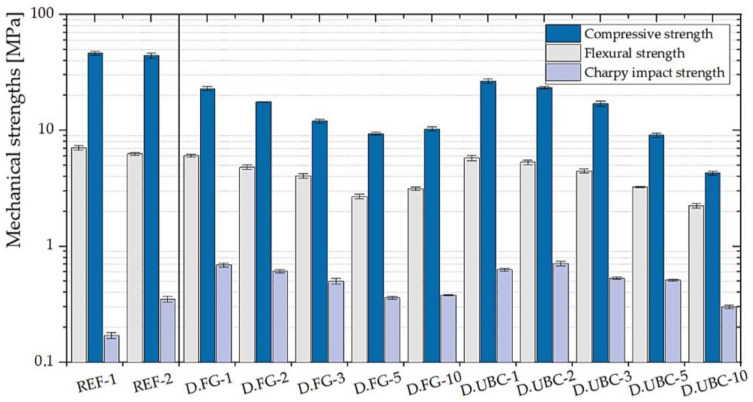
Mechanical properties of the geopolymer foams with the addition of various percentages of D.FG and D.UBC (1, 2, 3, 5, 10 wt.% of MK). Reference GPs (REF-1 and REF-2) are also shown.

The aluminum contents of the pyrolysis by-products D.FG and D.UBC are 32,204 and 40,198 ppm, respectively (Table 2). As shown in Figure 9, the mechanical strengths are better performed than the scraps of the screening processes. In this case, the impact strength of D.FG-1 is around four times higher than the reference sample REF-1 and two times more than REF-2. Moreover, also D.UBC-2 shows the same behavior with a σ_i_ of 0.71 MPa. This increase in performance is directly related to the aluminum content and finer-grained and more homogeneous particles of this kind of by-products.

#### 3.1.3. GFs with the Addition of the Aluminum-Rich By-Products of the Dust Abatement Systems (Cyclons)

**Figure 10:**
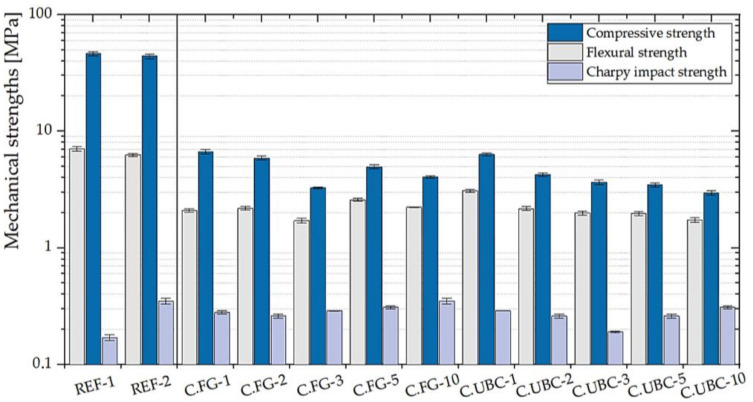
Mechanical properties of the geopolymer foams with the addition of various percentages of C.FG and C.UBC (1, 2, 3, 5, 10 wt.% of MK). Reference GPs (REF-1 and REF-2) are also shown.

C.FG and C.UBC raw materials have an aluminum content of 73,296 and 62,333 ppm. It is observable a conspicuous decrease of the flexural and compressive strengths (Figure 10) against the reference materials (REF-1, and REF-2), and also the impact strength compared to the standard with chopped carbon fibers, being the aluminum content around two times the one within geopolymers synthesized by the foaming agents D.FG—D.UBC.

#### 3.1.4. GFs with the Addition of the Aluminum-Rich By-Products of the Fusion Processes

**Figure 11:**
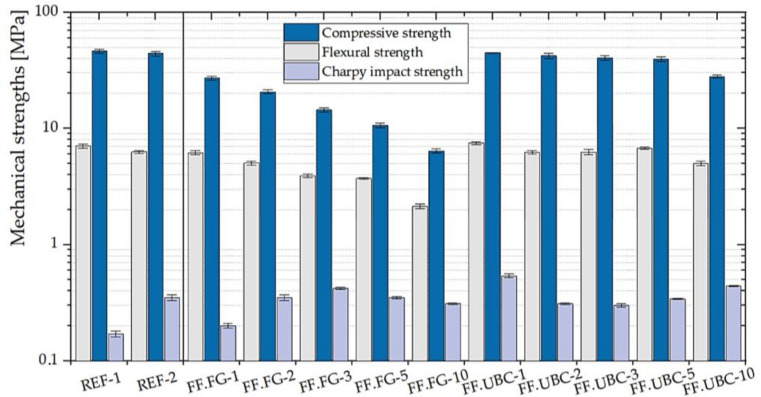
Mechanical properties of the geopolymer foams with the addition of various percentages of FF.FG and FF.UB (1, 2, 3, 5, 10 wt.% of MK). Reference GPs (REF-1 and REF-2) are also shown.

The best mechanical performances for the geopolymers obtained with the addition of the by-products of the fusion processes (Figure 11) are found in FF.UBC where compressive, flexural, and Charpy impact strengths are almost similar to the reference samples. In particular, FF.UBC-1 is the best GF in term of mechanical performance with σ_f_ = 7.48 ± 0.22 MPa; σ_c_ = 44.67 ± 0.31 MPa; σ_i_ = 0.54 ± 0.02 MPa. We can conclude that FF.UBC slag, having the lowest aluminum content (6636 ppm) is the most suitable by-product to be trapped into the geopolymeric structure keeping unchanged the fundamental mechanical properties of the reference geopolymers.

### 3.2. Densities versus Thermal Conductivity, Diffusivity, and Specific Heat

The density (ρ) and the thermal conductivity (λ), diffusivity (α), and specific heat ($C_{p}$) of the obtained geopolymer foams are reported in Table 5. A clear relationship between the density and the represented thermal properties can be observed.

**Figure 12:**
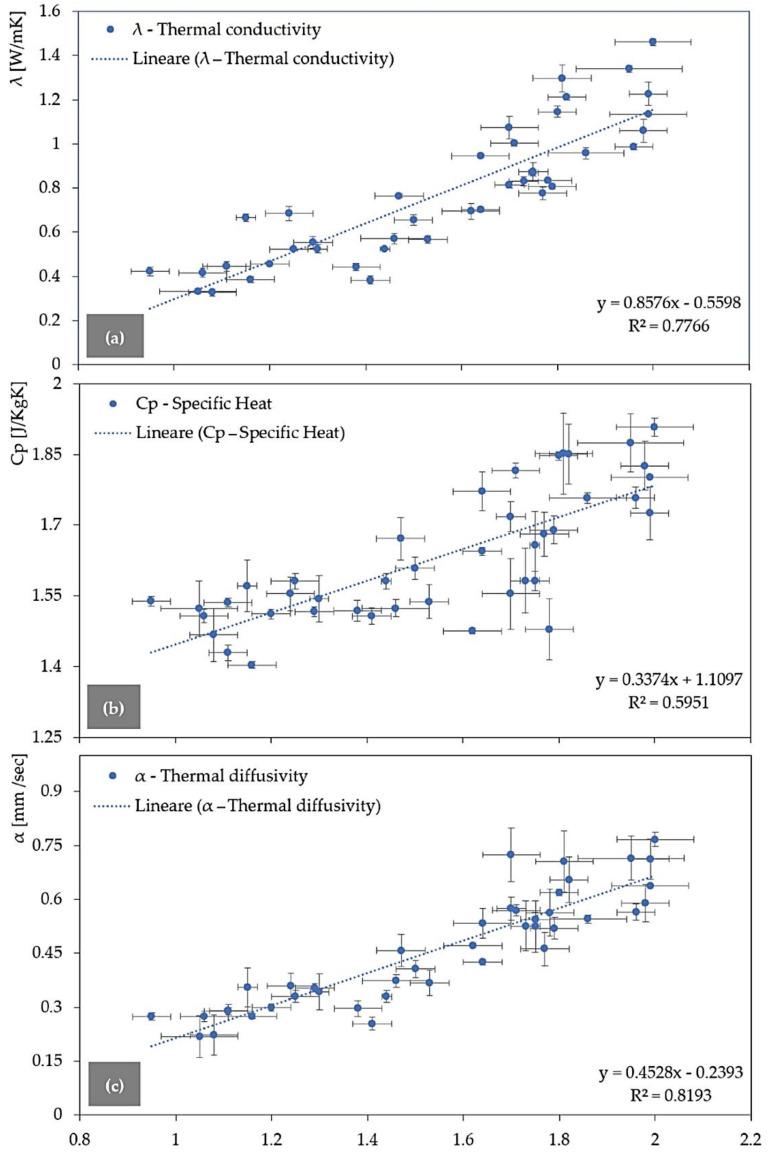
Thermal conductivity (**a**), specific heat (**b**), and thermal diffusivity (**c**) versus density for all the obtained geopolymer foams.

The linear regression of λ with ρ shows a R^2^ of 0.7766 (Figure 12a), so the thermal conductivity depends on the density of the geopolymers. Moreover, also $C_{p}$ (Figure 12b) and α (Figure 12c) are strongly related to the density with R^2^ of 0.5951 and 0.8193, respectively. For low densities, the porosity of the GFs increases, and consequently λ, Cp, and α significantly decrease. Definitively, the lower densities of these materials are a great advantage compared to the traditional building materials such as Portland cement. They are lightweight materials, and the thermal insulation properties are better performed. $\lambda ,{\;C}_{p}\;\mathrm{and}\;\alpha$ decrease by adding the industrial by-products which act as foaming agents.

REF-1 and REF-2, with a density of 1.81 ± 0.06, and 2 ± 0.08 g/cc show a λ of 1.2981 ± 0.0606, and 1.4607 ± 0.0167 W/mK, a $C_{p}$ of 1.8518 ± 0.0855, and 1.9078 ± 0.0194 J/KgK, an α of 0.7056 ± 0.0295, and 0.7667 ± 0.0124 mm^2^/sec, respectively. The higher values in the REF-2 are due to the chopped carbon fibers (CFs), which improve the mechanical properties, but on the other hand, increase the thermal properties by around 5–10%.

**Figure 13:**
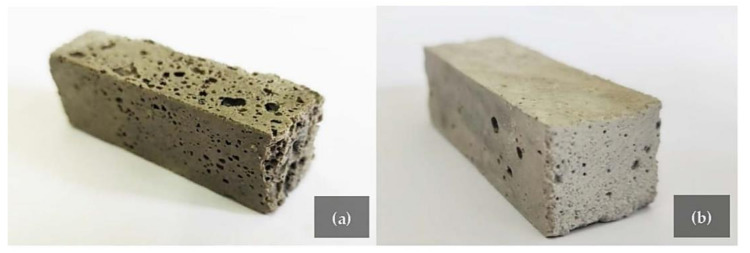
GFs obtained from C.FG-3 (**a**) and C.UBC-10 (**b**) represent the ones with the lowest thermal conductivity.

The densities decrease because of the foaming agents and range from 0.95 ± 0.04 g/cc (C.UBC-5) up to 1.99 ± 0.08 g/cc (D.FG-1). The lowest thermal conductivity (Table 5) was measured with the industrial by-products C.FG and C.UBC from the dust abatement collectors (cyclons). The geopolymer foam C.FG-3 (Figure 13a) recorded a thermal conductivity of 0.3306 ± 0.0069 W/mK and a density of 1.05 ± 0.08 g/cc. C.UBC-10 (Figure 13b) has an even lower λ of 0.3265 ± 0.0150 W/mK, and a density of 1.08 ± 0.05 g/cc.

### 3.3. Classification of the GFs

**Figure 14:**
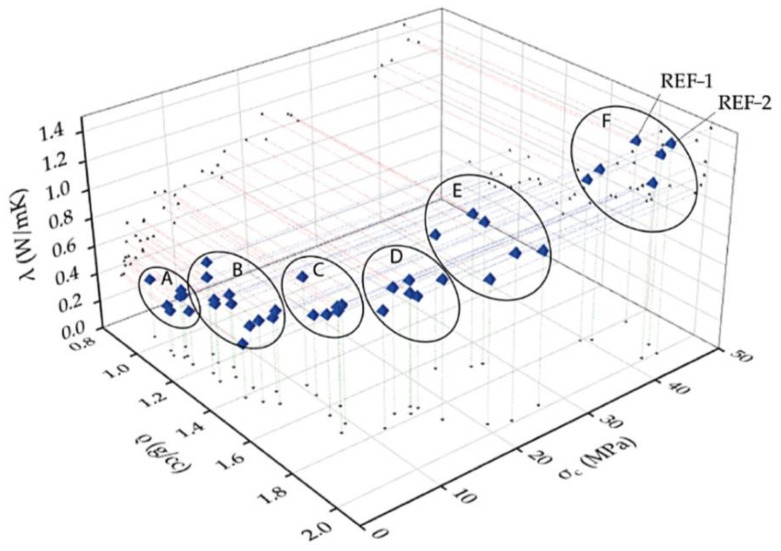
3D scatter plot of the density (ρ), compressive strength ($\sigma_{c}$) and thermal conductivity (λ) of the geopolymer foams (GFs).

The GFs were classified into six groups following the physical parameter of density versus compressive strength and thermal conductivity (Figure 14) to highlight which material has the best thermal insulation and mechanical properties.

Group A shows the lowest thermal conductivity values and the lowest densities from 0.95 to 1.16 g/cc. This population of data shows relatively low $\sigma_{c}$ ranging between 2.96 and 4.05 MPa. Group B has relatively higher densities than group A and, consequently, higher thermal conductivities. The compressive strengths are slightly higher, with an average value at around 5 MPa. Group C is characterized by $\sigma_{c}$ at around 10 MPa and λ that corresponds to 0.7 W/mK. The compressive strength of Group D range between 10 and 20 MPa, with thermal conductivity with an average value of 0.9 W/mK and a mean density of around 1.8 g/cc. Group E (density between 1.6 and 2 g/cc) is between 20 and 30 MPa for the compressive strength, with thermal conductivity of 1.1 W/mK. Finally, group F exhibits similar performance as the reference standard geopolymers (REF-1 and REF-2) concerning mechanical and thermal properties thanks to its higher density. The group F population shows a density between 1.8 and 2.0 g/cc, a mean λ of 1.3 W/mK, and mean $\sigma_{c}$ of around 42 MPa.

## 4. Conclusions

The present study deals with the mechanical (flexural, compressive, Charpy impact strengths) and thermal (thermal conductivity, specific heat, thermal diffusivity) properties of GFs obtained by adding aluminum-rich by-products of the secondary aluminum industry. According to the European Regulations, these industrial by-products cannot be disposed to landfills because they are classified as special hazardous wastes which can develop flammable gases and form explosive mixtures with air. The hazard mainly comes from hydrogen production due to metallic aluminum oxidation. Nevertheless, if the reaction producing hydrogen occurs when geopolymers are synthesized, the by-product themselves undergo a chemical neutralization, and the hydrogen-rich gas is used as foaming agents modifying the structure of standard geopolymers (REF-1, REF-2).

In particular, the work highlight that FF.UBC by-product coming from the fusion processes of the secondary aluminum industry is the most suitable material to improve the mechanical properties of geopolymers compared to REF-1 and REF-2, and it, therefore, is the appropriate raw material to foam lightweight geopolymers. In addition, significant decreases in thermal conductivity, specific heat, and thermal diffusivity, thus emphasizing good thermal insulation properties, are observed in the GFs doped with by-products C.FG and C.UBC from the dust abatement (cyclons) processes of the secondary aluminum industry.

The study unravels that using geopolymer foams as an alternative building material finds a compromise to balance the mechanical and thermal properties and guarantee the usability of the composite materials. For this reason, future studies will focus on mixing the three by-products (FF.UBC, C.FG, C.UBC), maintaining good mechanical performance for building material, and giving to GFs excellent thermal insulation properties those characterizing groups A-D of geopolymer foams with thermal conductivity ≤ 0.9 W/mK.

Accordingly, the final remarks are addressed to (i) recovery and process several by-products of the secondary aluminum industry, most of them not suitable to be disposed of in landfills; (ii) development of building materials with good mechanical and thermal insulation properties trapping the hazardous industrial by-products through the synthesis of GFs; (iii) reuse of the industrial by-products as a resource for new technological materials combining environmental sustainability and safety in the secondary aluminum industry workplaces, in the framework of a circular economy.

## Figures and Tables

**Table 1:** Chemical composition of the metakaolin (MK), silica sand (SA), and chopped carbon fibers (CFs).

	ρ (g/cc)	SiO_2_ (wt. %)	Al_2_O_3_ (wt.%)	TiO_2_ (wt.%)	Fe_2_O_3_ (wt.%)	K_2_O (wt.%)	MgO (wt.%)	CaO (wt.%)	C (wt.%)
MK	1.95	54.1	40.1	1.80	1.10	0.80	0.18	0.13	-
SA	2.65	99.4	-	-	0.04	-	-	-	-
CFs	1.8	-	-	-	-	-	-	-	>95%

**Table 2:** Density (ρ; g/cc) and metal contents (ppm) of by-products of the secondary aluminum industry. V.FG and V.UBC: screening process; D.FG and D.UBC: pyrolysis process; C.FG and C.UBC: dust abatement process; FF.FG, and FF.UBC: fusion process. The hazard classes of the dangerous substances were identified according to European regulations.

	V.FG	V.UBC	D.FG	D.UBC	C.FG	C.UBC	FF.FG	FF.UBC	Classification (CE Reg. 1272/2008)	HP (CE Reg. 1357/2014)
ρ (g/cc)	2.87 ± 0.01	2.69 ± 0.003	2.55 ± 0.09	2.58 ± 0.09	2.34 ± 0.04	2.40 ± 0.09	2.47 ± 0.11	2.52 ± 0.05
Al (ppm)	125,468	180,638	32,204	40,198	73,296	62,333	14,549	6636	H314	50,000 HP8
Sb (ppm)	<5	<5	6	<5	<5	<5	7.0	11	H314 H314 H411	10,000 HP4 50,000 HP8 250,000 HP 14
As (ppm)	<5	<5	<5	<5	<5	<5	<5	<5	H301 H331 H350 H400 H410	50,000 HP6 32,500 HP6 1000 HP7 250,000 HP14 250,000 HP14
B (ppm)	36.2	11.1	128.8	137.9	62.4	70.9	77	59	H360FD	3000 HP10
Cd (ppm)	36.2	7.4	25.8	46.1	27.6	70.6	91.3	36.3	H372 H330 H350 H361 H341	10,000 HP 5 1000 HP 6 1000 HP 7 30,000 HP 10 10,000 HP 11
Co (ppm)	28.5	<5	9.7	31.3	12.3	103.8	<5	<5	H317; H334	100,000 HP13
Cr^6+^ (ppm)	<0.5	<0.5	<0.5	<0.5	<0.5	<0.5	<0.5	<0.5	H340 H361f H317; H334 H350 H302 H410 H335; H372	1000 HP 11 30,000 HP 10 100,000 HP 13 1000 HP7 250,000 HP6 250,000 HP 14 10,000 HP 5
Cr (ppm)	49.5	107.9	182.3	34.7	326.5	222	94.8	30.3	-	-
Mn (ppm)	930.5	2891	302.4	585.6	671.6	717.8	129.6	29.5	H301; H302; H332H373	50,000 HP6 100,000 HP5
Mo (ppm)	<5	5	<5	<5	<5	<5	<5	<5	H315; H319 H351	200,000 HP 4 10,000 HP 7
Ni (ppm)	488.7	26.7	66.6	49.8	119.1	134.6	21.5	<5	H315 H301; H331 H350i H360D H341 H317; H334 H400 H411	200,000 HP 4 32,500 HP 6 1000 HP 7 3000 HP 10 10,000 HP 11 100,000 HP 13 250,000 HP 14 250,000 HP 14
Pb (ppm)	266.1	54.1	2756.4	787.8	3998.0	1378.0	369.5	165.6	H373 H360Df H410 H332	100,000 HP 5 3000 HP 10 250,000 HP 14 225,000 HP 6
Cu (ppm)	2710.3	1287.5	2045.9	640.2	2891.6	744.4	201.2	79.3	H315; H319 H302 H400 H410	200,000 HP 4 250,000 HP 6 250,000 HP 14 250,000 HP 14
Se (ppm)	<5	<5	<5	<5	<5	<5	78.8	20	H373H301; H331	100,000 HP5 32,500 HP6
Sn (ppm)	102.8	52.4	91.7	15.5	116.2	194.7	39.9	7.2	H314 H412	50,000 HP 8 250,000 HP 14
V (ppm)	29.6	56.4	24.6	12.6	23.8	23.7	<5.0	<5.0	H318 H335 H372 H300; H301; H302; H332 H341 H411	10,000 HP4 200,000 HP 5 10,000 HP 5 1000 HP 6 10,000 HP 11 250,000 HP 14
Zn (ppm)	14,539.0	3501.0	4774.2	3977.2	10,109.6	13,937.9	2028.6	689.4	H302 H315; H319 H335 H400 H410	250,000 HP 6 200,000 HP 4 200,000 HP 5 250,000 HP 14 250,000 HP 14

**Table 3:** The ratio of the main components used to synthesize the geopolymer foams with respect to MK content.

By Weight Ratio (-)				
Metakaolin (MK)	Alkaline Activator (A)	Carbon Fibers (CFs)	Silica Sand (SA)	Industrial By-Products (V.FG, V.UBC, D.FG, D.UBC, C.FG, C.UBC, FF.FG, FF.UBC)
1	0.9 MK	0.01 MK	1 MK	0.01 MK 0.02 MK 0.03 MK 0.05 MK 0.1 MK

**Table 4:** Summary of the mechanical properties (bending strength, $\sigma_{f}$; compressive strength, $\sigma_{c};$ impact strength, $\sigma_{i}$) of the reference geopolymers (REF-1, REF-2), and geopolymers foamed by various percentages (1, 2, 3, 5, 10 wt.% with respect the total amount of MK) of by-products from the screening (V.FG, V.UBC), pyrolysis (D.FG, D.UBC), dust abatement (C.FG, C.UBC) and fusion (FF.FG, FF.UBC) processes.

Geopolymers		Three-Point Bending Strength	Compressive Strength	Charpy Impact Strength
	By-Products (wt.% of MK)	$\sigma_{f}\;(\mathrm{MPa})$	$\sigma_{c}\;(\mathrm{MPa})$	$\sigma_{i}\;(\mathrm{MPa})$
REF-1	-	7.04 ± 0.31	46.24 ± 1.84	0.17 ± 0.01
REF-2	-	6.25 ± 0.20	44.02 ± 2.08	0.35 ± 0.02
V.FG-	1	4.41 ± 0.11	16.56 ± 0.71	0.32 ± 0.01
	2	3.87 ± 0.19	15.75 ± 0.85	0.44 ± 0.01
	3	3.61 ± 0.17	10.98 ± 0.54	0.35 ± 0.02
	5	2.62 ± 0.12	7.44 ± 0.34	0.28 ± 0.01
	10	1.78 ± 0.08	5.73 ± 0.13	0.29 ± 0.01
V.UBC-	1	4.25 ± 0.13	8.08 ± 0.22	0.39 ± 0.01
	2	3.05 ± 0.13	5.96 ± 0.26	0.36 ± 0.02
	3	2.26 ± 0.11	5.19 ± 0.24	0.30 ± 0.01
	5	2.59 ± 0.08	3.95 ± 0.08	0.30 ± 0.01
	10	1.55 ± 0.08	3.56 ± 0.05	0.26 ± 0.01
D.FG-	1	6.04 ± 0.19	22.96 ± 0.96	0.69 ± 0.03
	2	4.81 ± 0.22	17.57 ± 0.06	0.61 ± 0.02
	3	4.05 ± 0.20	12.00 ± 0.50	0.50 ± 0.03
	5	2.69 ± 0.12	9.39 ± 0.32	0.36 ± 0.01
	10	3.14 ± 0.12	10.28 ± 0.40	0.38 ± 0.004
D.UBC-	1	5.78 ± 0.28	26.58 ± 1.32	0.63 ± 0.02
	2	5.31 ± 0.24	23.35 ± 0.63	0.71 ± 0.03
	3	4.46 ± 0.19	16.99 ± 0.80	0.53 ± 0.01
	5	3.25 ± 0.05	9.08 ± 0.36	0.51 ± 0.01
	10	2.24 ± 0.11	4.27 ± 0.19	0.30 ± 0.01
C.FG-	1	2.09 ± 0.08	6.67 ± 0.30	0.28 ± 0.01
	2	2.18 ± 0.08	5.90 ± 0.25	0.26 ± 0.01
	3	1.71 ± 0.08	3.27 ± 0.06	0.29 ± 0.002
	5	2.58 ± 0.07	4.94 ± 0.19	0.31 ± 0.01
	10	2.23 ± 0.01	4.05 ± 0.09	0.35 ± 0.02
C.UBC-	1	3.09 ± 0.09	6.35 ± 0.18	0.29 ± 0.001
	2	2.17 ± 0.09	4.24 ± 0.17	0.26 ± 0.01
	3	1.99 ± 0.08	3.66 ± 0.16	0.19 ± 0.003
	5	1.96 ± 0.07	3.46 ± 0.13	0.26 ± 0.01
	10	1.74 ± 0.08	2.96 ± 0.13	0.31 ± 0.01
FF.FG-	1	6.18 ± 0.25	27.03 ± 1.03	0.20 ± 0.01
	2	5.03 ± 0.20	20.60 ± 0.88	0.35 ± 0.02
	3	3.91 ± 0.15	14.44 ± 0.65	0.42 ± 0.01
	5	3.71 ± 0.07	10.64 ± 0.49	0.35 ± 0.01
	10	2.15 ± 0.10	6.40 ± 0.27	0.31 ± 0.005
FF.UBC-	1	7.48 ± 0.22	44.67 ± 0.31	0.54 ± 0.02
	2	6.21 ± 0.23	42.05 ± 2.07	0.31 ± 0.004
	3	6.24 ± 0.31	40.53 ± 1.85	0.30 ± 0.01
	5	6.77 ± 0.16	39.47 ± 1.88	0.34 ± 0.004
	10	5.01 ± 0.23	27.92 ± 0.84	0.44 ± 0.003

**Table 5:** Summary of density (ρ, g/cc) and thermal properties (thermal conductivity, λ; specific heat, $C_{p};$ diffusivity, α) of the synthesized geopolymer foams, by adding (1, 2, 3, 5, 10 wt.% of MK) the by-products from the screening (V.FG and V.UBC), pyrolysis (D.FG and D.UBC), abatement dust (C.FG and C.UBC) and fusion (FF.FG and FF.UBC) processes.

**Geopolymer**	By-Products (wt.% of MK)	ρ **(g/cc)**	λ (W/mK)	$C_{p}$ (J/KgK)	α (mm^2^/sec)
REF-1	-	1.81 ± 0.06	1.2981 ± 0.0606	1.8518 ± 0.0855	0.7056 ± 0.0295
REF-2	-	2.00 ± 0.08	1.4607 ± 0.0167	1.9078 ± 0.0194	0.7667 ± 0.0124
V.FG-	1	1.75 ± 0.03	0.8740 ± 0.0414	1.5828 ± 0.0206	0.5447 ± 0.0256
	2	1.78 ± 0.05	0.8330 ± 0.0050	1.4794 ± 0.0649	0.5639 ± 0.0216
	3	1.62 ± 0.06	0.6947 ± 0.0345	1.4758 ± 0.0065	0.4709 ± 0.021
	5	1.44 ± 0.01	0.5239 ± 0.0039	1.5815 ± 0.0166	0.3302 ± 0.0058
	10	1.30 ± 0.02	0.5249 ± 0.0190	1.5446 ± 0.0495	0.3426 ± 0.0145
V.UBC-	1	1.73 ± 0.03	0.8304 ± 0.0199	1.5828 ± 0.0692	0.5264 ± 0.0277
	2	1.38 ± 0.05	0.4424 ± 0.0183	1.5195 ± 0.0219	0.2969 ± 0.0139
	3	1.46 ± 0.07	0.5723 ± 0.0237	1.5245 ± 0.0179	0.3735 ± 0.0114
	5	1.29 ± 0.04	0.5533 ± 0.0273	1.5168 ± 0.0108	0.3544 ± 0.0186
	10	1.16 ± 0.05	0.3864 ± 0.0131	1.4043 ± 0.0073	0.2752 ± 0.0079
D.FG-	1	1.99 ± 0.08	1.1351 ± 0.0065	1.8014 ± 0.0016	0.6383 ± 0.0093
	2	1.86 ± 0.08	0.9585 ± 0.0255	1.7580 ± 0.0114	0.5453 ± 0.0181
	3	1.79 ± 0.05	0.8056 ± 0.0091	1.6898 ± 0.0293	0.5203 ± 0.1047
	5	1.70 ± 0.03	0.8150 ± 0.0134	1.7186 ± 0.0318	0.5745 ± 0.0164
	10	1.53 ± 0.04	0.5666 ± 0.0164	1.5385 ± 0.0357	0.3683 ± 0.0026
D.UBC-	1	1.70 ± 0.06	1.0742 ± 0.0510	1.5547 ± 0.0751	0.7246 ± 0.0319
	2	1.64 ± 0.06	0.9446 ± 0.0073	1.7715 ± 0.0411	0.5336 ± 0.0164
	3	1.77 ± 0.05	0.7761 ± 0.0291	1.6812 ± 0.0466	0.4624 ± 0.0231
	5	1.64 ± 0.04	0.7012 ± 0.0097	1.6448 ± 0.0090	0.4263 ± 0.0037
	10	1.24 ± 0.05	0.6841 ± 0.0320	1.5549 ± 0.0353	0.3597 ± 0.0158
C.FG-	1	1.25 ± 0.05	0.5222 ± 0.0038	1.5815 ± 0.0166	0.3302 ± 0.0058
	2	1.20 ± 0.04	0.4568 ± 0.0074	1.5125 ± 0.0102	0.2999 ± 0.0066
	3	1.05 ± 0.08	0.3306 ± 0.0069	1.5235 ± 0.0583	0.2194 ± 0.0008
	5	1.06 ± 0.05	0.4154 ± 0.0190	1.5079 ± 0.0145	0.2754 ± 0.0099
	10	1.11 ± 0.04	0.4444 ± 0.0212	1.4302 ± 0.0167	0.2903 ± 0.0135
C.UBC-	1	1.50 ± 0.04	0.6539 ± 0.0239	1.6092 ± 0.0230	0.4064 ± 0.0150
	2	1.41 ± 0.04	0.3829 ± 0.0191	1.5076 ± 0.0173	0.2542 ± 0.0218
	3	1.11 ± 0.05	0.4443 ± 0.0076	1.5359 ± 0.0096	0.2892 ± 0.0032
	5	0.95 ± 0.04	0.4217 ± 0.0205	1.5390 ± 0.0103	0.2741 ± 0.0132
	10	1.08 ± 0.05	0.3265 ± 0.0150	1.4678 ± 0.0558	0.2220 ± 0.0113
FF.FG-	1	1.98 ± 0.05	1.0599 ± 0.0528	1.8263 ± 0.0520	0.5892 ± 0.0288
	2	1.96 ± 0.04	0.9873 ± 0.0130	1.7583 ± 0.0226	0.5643 ± 0.0024
	3	1.75 ± 0.01	0.8694 ± 0.0134	1.6577 ± 0.0717	0.5250 ± 0.0147
	5	1.47 ± 0.05	0.7647 ± 0.0071	1.6716 ± 0.0445	0.4577 ± 0.0112
	10	1.15 ± 0.02	0.6655 ± 0.0178	1.5719 ± 0.0540	0.3564 ± 0.0094
FF.UBC-	1	1.95 ± 0.11	1.3399 ± 0.0153	1.8743 ± 0.0613	0.7152 ± 0.0152
	2	1.99 ± 0.04	1.2267 ± 0.0531	1.7256 ± 0.0564	0.7127 ± 0.0656
	3	1.82 ± 0.04	1.2116 ± 0.0126	1.8506 ± 0.0628	0.6551 ± 0.0155
	5	1.80 ± 0.04	1.1462 ± 0.0245	1.8472 ± 0.0090	0.6197 ± 0.0168
	10	1.71 ± 0.05	1.0018 ± 0.0119	1.8159 ± 0.0154	0.5700 ± 0.0452
